# Epithelial changes with corneal punctate epitheliopathy in type 2 diabetes mellitus and their correlation with time to healing

**DOI:** 10.1186/s12886-017-0645-6

**Published:** 2018-01-04

**Authors:** Jing-hao Qu, Li Li, Lei Tian, Xiao-yu Zhang, Ravi Thomas, Xu-guang Sun

**Affiliations:** 1 Beijing Institute of Ophthalmology, Beijing Tongren Eye Center, Beijing Tongren Hospital, Capital Medical University; Beijing Ophthalmology & Visual Sciences Key Laboratory, Beijing, 100730 China; 2 The First Affiliated Hospital of Xi’an Jiaotong University, Xi’an, 710061 China; 3 Queensland Eye Institute, Brisbane, Australia; 4 University of Queensland, Brisbane, Australia

**Keywords:** Type 2 diabetes mellitus, Corneal punctate epitheliopathy, Langerhans cell, Subbasal nerve plexus, Basal epithelial cell

## Abstract

**Background:**

To study basal epithelial cell (BEC), sub-basal nerve plexus (SBN) and Langerhans cell (LC) density in patients with type 2 diabetes mellitus (T2DM) with corneal punctate epitheliopathy (CPE) and to assess their association with time to healing of CPE.

**Methods:**

Retrospective study of in vivo confocal microscopy (IVCM) in 160 eyes from 160 patients with T2DM diagnosed with CPE due to a single cause. Key exclusion criteria included multiple-causes for CPE or treatment with autologous serum. A total of 149 eyes from 149 gender- age- and aetiolgy-matched patients with CPE without T2DM comprised the control group. Electronic records were reviewed for demographic features, history of T2DM and aetiology of CPE. Density of BEC, SBN and LC were compared between the two groups.

**Results:**

The healing time in days for CPE with different aetiologies in the T2DM and control groups were as follows: dry eye (21.56 ± 2.41; 7.00 ± 2.19; *P* = 0.001); meibomian gland dysfunction (26.42 ± 6.04; 9.21 ± 2.55; P = 0.001); cataract extraction (38.00 ± 19.62; 25.83 ± 11.49; *P* = 0.043); drug induced (53.19 ± 18.83; 41.86 ± 23.87; *P* = 0.018) and exposure (38.25 ± 14.13; 29.00 ± 13.67; *P* = 0.026). LC density was 38.70 ± 9.65 cells/mm^2^ in the T2DM group comparedwith 25.53 ± 3.54 cells/mm^2^ in the controls (*P* = 0.001). SBN density was 11.76 ± 1.69 mm/mm^2^ in the T2DM group compared with 20.92 ± 1.43 mm/mm^2^ in the controls (*P* = 0.001). BEC density in the T2DM group was 4982 ± 1178 cells/mm^2^ compared with 5739 ± 394 cells/mm^2^ in the control group (*P* = 0.018). Age and duration of T2DM had no relationship with healing time (multiple linear regression, *P* = 0.618; *P* = 0.787). The density of LC in the T2DM group showed a negative correlation with SBN density (*r* = 0.350; *R*^2^ = 0.1225; *P* = 0.034). The density of SBN in the T2DM group showed a positive correlation with BEC density (*r* = 0.427; *R*^2^ = 0.1823; *P* = 0.008). The density of BEC in the T2DM group showed a negative correlation with healing time (*r* = 0.931; *R*^2^ = 0.8668; *P* = 0.001).

**Conclusions:**

Utilising IVCM, we have demonstrated increased LC and decreased SBN in patients with T2DM and CPE. Both may be related to lower BEC density and nuclei enhanced reflection. Furthermore, decreased BEC density may lead to delay in cornea epithelium healing in the T2DM group comparedwith controls. An immune-mediated response may play a role in delayed wound closure in patients with T2DM.

## Background

An estimated 415 million people were reported to have diabetes mellitus in 2017 and this figure is projected to rise to 642 million by 2040, an increase of 55% [1]. Type 2 diabetes mellitus (T2DM) is closely related to modern lifestyle, ageing and obesity and is associated with numerous systemic and ocular complications. While diabetic retinopathy (DR) is the commonest ocular complication and a leading cause of blindness worldwide, the ocular surface can also be affected. T2DM affects the morphological, physiological, metabolic and clinical state of the cornea, and microstructural changes can still exist even with a normal slit lamp examination and can predispose patients to disease [2]. The prevalence of corneal abnormalities such as punctate keratitis, recurrent corneal erosion syndrome, persistent epithelial defects, reduced corneal sensitivity and endothelial damage is increased in diabetics [3, 4].

The corneal epithelium is an important defensive barrier of the ocular surface [5]. Patients with T2DM have epithelial basement membrane abnormalities in the nervous system, skin, kidney, retina and cornea [6–9]. The effects of T2DM impact epithelial basal cells, the epithelial basement membrane as well as sub-basal nerve plexus (SBN) and can lead to epitheliopathy and corneal adhesion disorders [10]. Basement membrane changes in T2DM can affect the corneal epithelium and predispose to infections. Such changes include a reduction in corneal epithelial basal cells (BEC) as well as an increase in thickness and irregularity of the corneal epithelial basement membrane [7, 11]. T2DM affects the corneal SBN leading to a reduction in SBN density [12]. Furthermore, it is of interest that Cornea confocal microscopy (CCM) has revealed an increase in the number of highly reflective cells, ‘presumably dendritic cells’, in the cornea of patients with diabetes [13]. These cells were subsequently identified as Langerhans cell (LC) [14].

In vivo confocal microscopy (IVCM) has become the standard tool for assessing the living cornea at a cellular level both in diseases states and in healthy subjects [15]. The instrument has excellent repeatability and reproducibility in the evaluation of the corneal epithelium, sub-basal nerve plexus (SBN) and LC density, in both healthy controls and in people with T2DM [16–18].

Corneal punctuate epitheliopathy (CPE) refers to a group of diseases that show dot-like staining of the corneal epithelium. Patients complain of sharp pain, tearing, redness, foreign body sensation, photophobia and decreased vision. The manifestations of CPE include an increase in corneal epithelial permeability, epithelial edema and dot-like staining. If untreated or incorrectly treated,CPE can lead on to corneal ulceration [10].

The objective of this study was to examineBEC, SBN and LC density in CPE in patients with T2DM and report their association with time to healing of CPE.

## Methods

A total of 928 patients received a diagnosis of CPE during the study period fromNovember 2010 to October 2015. Patientsfor whom CPE was attributed to multiple factors (a single causes could not be established, single cause include: dry eye; MGD; cataract extraction; drug; exposure) and who had undergone previous corneal or ocular surgery, had any ocular pathology other than diabetic retinopathy and keratopathy were excluded. One eye of each patient was selected for analysis, and in patients with bilateral CPE, the most severely affected eye was selected. The Ethics Committee of the Tongren Hospital (Beijing, China) approved the study.

In all, 160 eyesfrom 160 patients with T2DMand CPE were eligible for inclusion in the study. The control group consisted of 149 eyesfrom 149 gender-, age- and aetiology-matched patients with CPE without T2DM who were seen during the study period.

All patients underwent visual acuity testing as well as slit lamp biomicroscopic examination with fluorescein staining. A corneal specialist made the diagnosis of CPE based on a combination of symptoms and clinical signs and determined theaetiology. The symptoms included a combination of sharp pain, tearing, redness, foreign body sensation, photophobia and decreased vision combined with more than 5 spots of epithelial dots that stained with fluorescein [10]. Other data collected included age, sex, affected eye, worse affected eye, history of surgery, use of ocular medication, presence of MGD and T2DM. T2DM was diagnosed based on the standards of medical care in diabetes [19].

CPE was divided into the following aetiological categories:

1. Dry eye related: normal eye lid margin with tear film break up time (TFBUT) <5 s, Schirmer1test < 5 mm/5 min [20];
2. Meibomian gland disease (MGD) related: patients with lid signs of MGD [21];
3. Cataract extraction related: CPE occurring within 2 weeks of surgery in the absence of medication use (as detailed below), dry eye or MGD.
4. Drug related: CPE in those using eye drops (tobramycin or dexamethasone) or ganciclovir gel for more than 1 month and absence of dry eye and MGD;
5. Exposure related: CPE resulting from corneal exposure with corneal staining located inferiorly in the area of exposure, in the absence of dry eye and MGD.

All patients underwent IVCM in both eyes using the Heidelberg Retina Tomograph III Rostock Cornea Module (HRT III RCM; Heidelberg Engineering GmbH, Heidelberg, Germany). Genteal Gel (0.2% carbomer eye drops; Dr. Gerhard Mann, Chem.-Pharm, Fabrik GmbH) was applied in a disposable sterile polymethylmethacrylate cap (Tomo-Cap; Heidelberg Engineering GmbH)placed on the tip of the objective lens. A drop of local anaesthetic (0.4% tetracaine hydrochloride) was administered to both eyes and the subject was asked to fixate on a distant target before commencing scanning of the central cornea. A single experienced and masked examiner performed all scans. Section scans of the central cornea were recorded with the Heidelberg HRT-III microscope, using 384 × 384 pixels and a field of view of 400 × 400 μm^2^.

One randomly selected eye from each person in the control group was used for analysis. Three good quality images of the LC, SBN and BEC were selected and were used for image analysis by the examiner. The average of three measurements was used for further comparative analysis [22]. The basal epithelium was defined as the first three clear scans anterior to Bowman’s layer. BEC was measured manually using HRT III proprietary software.On average, three quality images of Bowman’s layer were used to quantify both Langerhans cell density and nerve fibre morphology in all patients and controls, and the average results of all these images were calculated. SBN density was defined as the total nerves lengths in units of mm/mm^2^ [23]. SBN was analysed in three selected high-quality images using NeuronJ software (Erik Meijering). NeuronJ is an ImageJ (National Institutes of Health, Bethesda, MD) plugin to facilitate the tracing and quantification of elongated image structures [24].

The therapy for CPE was standardized and included sodium hyaluronate eye drops and de-proteinized calf blood extract eye gel 4 times a day [25]. All patients were examined within 3-7 days of the initiation of treatment and at a similar interval thereafter untill healing was complete.

**Fig. 1:**
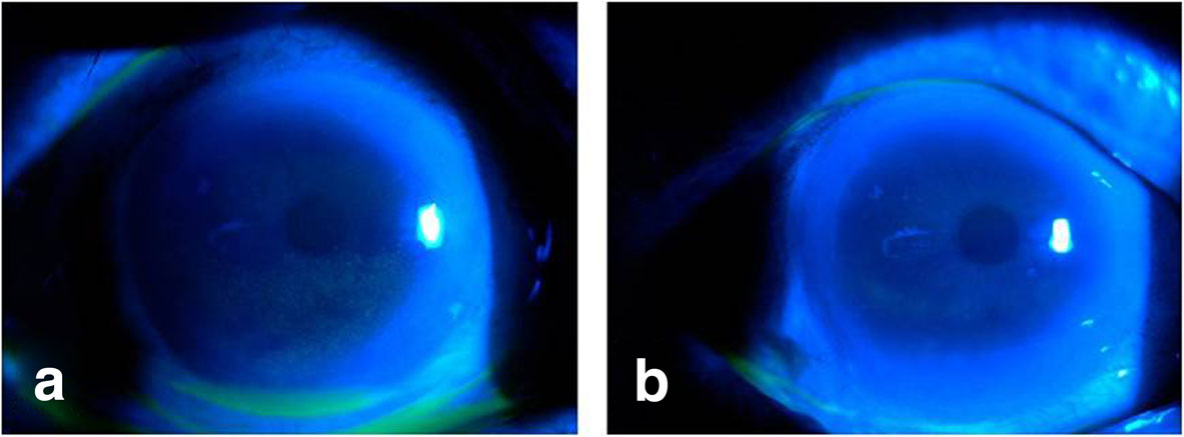
Pre and post treatment photograph of a patient with T2DM and CPE. **a** Pre-therapy corneal punctate epitheliopathy (corneal epithelial dot staining positive). **b** Post-therapy showing absence of fluorescein staining

The healing time was defined as the number of days from the onset of treatment to the day the corneal epithelial fluorescein staining became negative. An example of a patient with T2DM who developed CPE following cataract surgery is shown in Fig. 1. Corneal staining resolved 21 days after the initiation of treatment.

All statistical analyses were performed with SPSS (version 18.0). Chi-square analysis, two samples rank sum test, Pearson correlation and multiple linear regression were used. *P* < 0.05 was considered to be statistically significant.

## Results

**Fig. 2:**
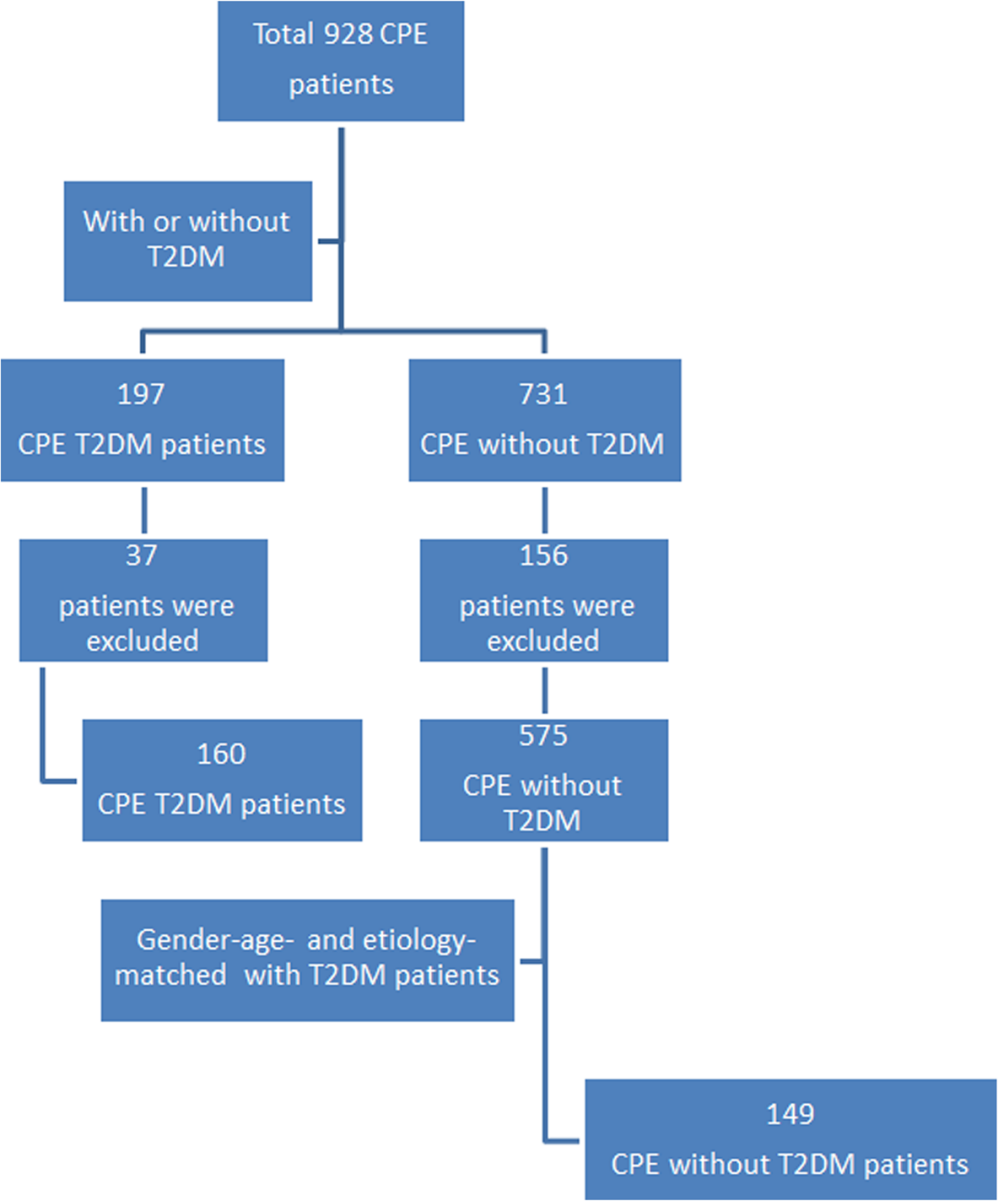
Number of patients included in the study (gender: χ2 analysis, *P* > 0.05; age: two samples rank sum test, *P* > 0.05)

Of the 928 patients diagnosed with CPE during the study period, 197 had T2DM. Thirty-seven patients with T2DM and 156 controls for whom a single aetiological category could not be identified as the cause for CPE were excluded. Included were160 patients (62 males; 98 females) and 149 controls (60 males; 89 females) with an average age of 59.8 ± 11.6 and 58.9 ± 14.9 years respectively (Fig. 2). The duration of T2DM was 13.4 ± 8.30 year (from 1 to 30 years).

The cause of CPE in the T2DM group were dry eye (40, 25.0%), meibomian gland dysfunction (40, 25.0%), cataract extraction (38, 23.8%), drug induced (21, 13.1%) and exposure (21, 13.1%). The control group was matched for gender, age andaetiology. The cause of CPE in the control group were dry eye (38, 25.5%), meibomian gland dysfunction (38, 25.5%), cataract extraction (34, 22.8%), drug induced (19, 12.8%) and exposure (20, 13.4%).

**Fig. 3:**
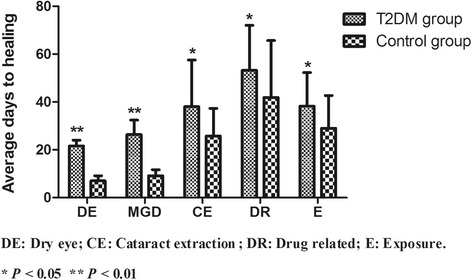
Healing timesfor CPE in patients with T2DM and controls. Healing times were significantly higher in theT2DM group irrespective of aetiology

The healing times for the different categories of CPE in the T2DM and control groups are shown in Table 1 and Fig. 3. The healing times were longer in T2DM patients across all aetiologic categories.

**Table 1:** Healing time of CPE in T2DM patients and controls

	Eyes	Days to healing (mean ± SD)	*P* values
Dry eye			
T2DM	40	21.56 ± 2.41	*P* = 0.001
Controls	35	7.00 ± 2.19	
MGD			
T2DM	42	26.42 ± 6.04	*P* = 0.001
Controls	37	9.21 ± 2.55	
Cataract extraction			
T2DM	38	38.00 ± 19.62	*P* = 0.043
Controls	35	25.83 ± 11.49	
Drug			
T2DM	20	53.19 ± 18.83	*P* = 0.018
Controls	21	41.86 ± 23.87	
Exposure			
T2DM	20	38.25 ± 14.13	*P* = 0.026
Controls	21	29.00 ± 13.67	
Total	309	25.97 ± 18.21	–

**Fig. 4:**
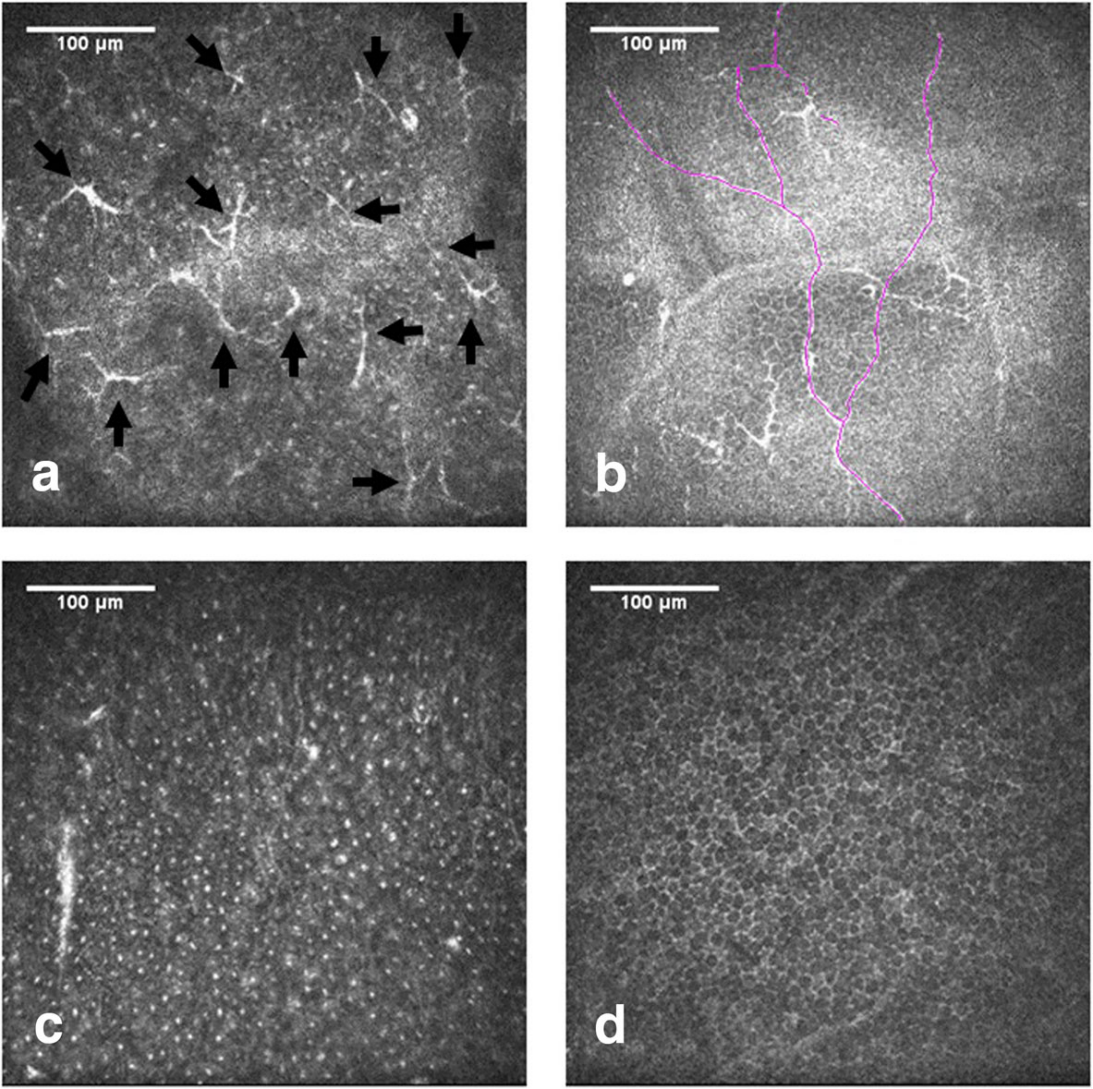
ICVM of basal epithelial cells in a patient with T2DM (**a**-**c**) and a control patient (**d**). Images from Bowman’s layer of the cornea with highly reflective Langerhans cell shown by black arrows (**a**); representative image showing decreased SBN density in T2DM (**b**); patient with T2DM showing lower basal epithelial cell density (**c**)

Results of the ICVM examination were shown in Table 2. The proportion of individuals with LC was significantly higher in the T2DM group (82.1%) compared with the control group (46.1%, *P* = 0.001). LC density was also significantly higher in diabetic patients compared with controls (38.70 ± 9.65 cells/mm^2^ vs 25.53 ± 3.54 cells/mm^2^, *P* = 0.001; Fig. 4a). Central corneal SBN density was 11.76 ± 1.69 mm/mm^2^ in T2DM vs 20.92 ± 1.43 mm/mm^2^ in controls (*P* = 0.001; Fig. 4b). BEC density in the T2DM group (4982 ± 1178 cells/mm^2^) was significantly lower than that in the control group (5739 ± 394 cells/mm^2^; *P* = 0.018) (Fig. 4c).

**Table 2:** LC density (cells/mm^2^), basal epithelial cell density (cells/mm^2^) and SBN density (mm/mm^2^) in T2DM and control group

	Age (mean ± SD)	LC density	SBN density	BCE density
Type 2 DM	59.8 ± 11.6	38.70 ± 9.65	11.76 ± 1.69	4982 ± 1178
Control	58.9 ± 14.9	25.53 ± 3.54	20.92 ± 1.43	5739 ± 394

LC density using two samples rank sum test *P* = 0.001; SBN density using two samples rank sum test *P* = 0.001; BCE density using two samples rank sum test *P* = 0.018

**Fig. 5:**
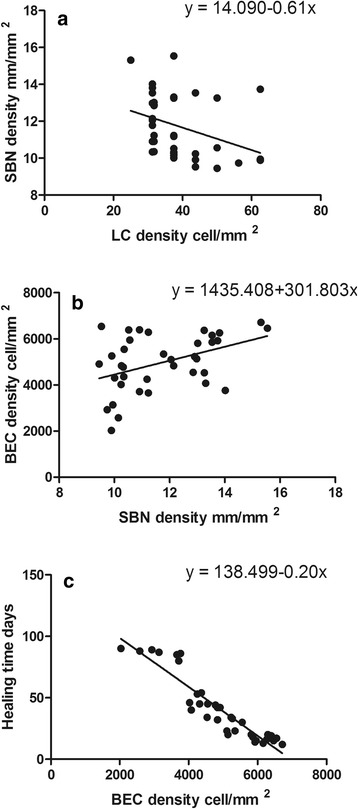
Correlation with healing time, LC, SBN and BEC. The density of LC in the T2DM group showed a negative correlation with SBN density (**a**); the density of SBN in the T2DM group showed a positive correlation with BEC density (**b**); the density of BEC in the T2DM group showed a negative correlation with healing time (**c**)

Age and duration of T2DM had no relationship with healing time (multiple linear regression, *P* = 0.618; *P* = 0.787). The density of LC in the T2DM group showed a negative correlation with SBN density (Fig. 5a; Pearson correlation *r* = 0.350; R2 = 0.1225; *P* = 0.034). There was no correlationbetween LC density and SBN density in the control group (*r* = 0.027; *P* = 0.913). The density of SBN in the T2DM group showed a positive correlation with BEC density (Fig. 5b; Pearson correlation *r* = 0.427; R2 = 0.1823; *P* = 0.008). There was no correlation between SBN density and BEC density in the control group (*r* = −0.104; *P* = 0.673). The density of BEC in the T2DM group showed a negative correlation with healing time (Fig. 5c; Pearson correlation *r* = 0.931; R2 = 0.8668; *P* = 0.001). There was no correlation between BEC density with healing time in the control group (*r* = −0.150; *P* = 0.540).

## Discussion

Age and duration of T2DM had no relationship with healing time. The key finding is that SBN density was decreased in patients with CPE and T2DM, while BEC density was also decreased and prolonged healing time. T2DM is one of the common causes of corneal nerve deficits and poor epithelium healing time [26]. The density of BEC in patients with CPE and T2DM was significantly lower than in the control group (*P* = 0.018; Table 2).This results is similar to that of Quadrado et al. [11] and Chang et al. [27] who reported lower BEC density in patients with T2DM. The density of BEC in our study was lower than previous reports that compared patients with T2DM with healthy controls, because our study mainly focused on patients with CPE. The metabolism of corneal epithelial (cell proliferation, differentiation, migration, and death) depends on corneal innervation, T2DM patients’ cornea decreased in SBN can result in the loss of BEC [10]. Corneal sensory nerves supply trophic neuropeptides, such as calcitonin gene-related peptide (CGRP). These compounds increase corneal epithelial cells’ proliferation and modulate epithelial cell differentiation and migration [11]. The dysfunction of BEC could lead to prolonged in healing time.

The total nerve length that used in our study was a reliable measure of corneal nerve density [28]. Ziegler et al. [29] and Zhivov et al. [30] reported the SBN density in patients with T2DM, to be 19.7 ± 7.5 mm/mm^2^ and 6.2 ± 4.4 mm/mm^2^, respectively. We also showed that SBN density was decreased in patients with CPE and T2DM. The SBN density in patients with CPE without T2DM was quite similar with the earlier study we mentioned above.

LC was mainly located in the central and peripheral of cornea [31, 32]. Previous studies have [33] reported LC was located beneath the basal epithelial cells. The density of LC in our T2DM group (38.70 ± 9.65 cells/mm^2^) and control group (25.53 ± 3.54 cells/mm^2^) differs from the results reported by Tavakoli et al. (17.73 ± 1.45 cells/mm^2^ verus 6.94 ± 1.45 cells/mm^2^). However, the results reported by Mastropasquaare the same as those of our control group (24 ± 10 cell/mm^2^) [34]. This is because HRT III has a higher resolution ratio for identifying LC when compared with the Tomey Confoscan corneal confocal microscope.

The density of BEC showed no relationship with healing time in the control group because the SBN density was normal in this group. In addition, the density of LC was increased in patients with CPE and T2DM. Tavakoli et al. [14] reported corneal nerve damage led to increased LC in T2DM patients. Immune mediated might contribution to corneal nerve damage in T2DM patients. In our study, the density of LC in the T2DM group showed a negative correlation with SBN density, providing support to Tavakoli’s hypothesis. This suggests that SBN along with LC may play a role in delayed wound closure in T2DM patients.

These data provide support for the role of IVCM in the microstructural evaluation of the corneal epithelium and the associated factors with CPE. However, there are several limitations to our study. IVCM images were only from the first patient visit, and there are no post-treatment images for comparison. In addition, the glycaemic control data was not collected in T2DM patients.

## Conclusions

In summary, these data provide support for a potential immune-mediated role in delayed wound healing seen in patients with T2DMand CPE. In the future, mechanistic studies are warranted to define the basis of this immune-mediated damage with the goal of developing therapeutic strategies to shorten the healing time.

## Abbreviations

BEC: Basal epithelial cell
CCM: Cornea confocal microscopy
CGRP: Calcitonin gene-related peptide
CPE: Corneal punctate epitheliopathy
DCs: Dendritic cells
IVCM: In vivo confocal microscopy
LC: Langerhans cell
MGD: Meibomian gland disease
SBN: Sub-basal nerve plexus
T2DM: Type 2 diabetes mellitus
TFBUT: Tear film break up time
